# Assessment of dimethyl sulphide odorous emissions during coal extraction process in Coal Mine Velenje

**DOI:** 10.1007/s10661-023-11755-z

**Published:** 2023-10-04

**Authors:** Gregor Uranjek, Milena Horvat, Radmila Milačič, Janez Rošer, Jože Kotnik

**Affiliations:** 1 Coal Mine Velenje, Partizanska 78, 3320 Velenje, Slovenia; 2 Jožef Stefan International Postgraduate School Ljubljana, Jamova 39, 1000 Ljubljana, Slovenia; 3 Department of Environmental Sciences, Jožef Stefan Institute, Jamova 39, 1000 Ljubljana, Slovenia; 4 Faculty of Natural Sciences and Engineering, Aškerčeva 12, 1000 Ljubljana, Slovenia

**Keywords:** Coal mine, Dimethyl sulphide, Odour, Coal gases, Mine ventilation, Dispersion modelling

## Abstract

Underground coal extraction at Coal Mine Velenje occasionally gives rise to odour complaints from local residents. This manuscript describes a robust quantification of odorous emissions of mine sources and a model-based analysis aimed to establish a better understanding of the sources, concentrations, dispersion, and possible control of odorous compounds during coal extraction process. Major odour sources during underground mining are released volatile sulphur compounds from coal seam that have characteristic malodours at extremely low concentrations at micrograms per cubic metre (μg/m^3^) levels. Analysis of 1028 gas samples taken over a 6-year period (2008–2013) reveals that dimethyl sulphide ((CH_3_)_2_S) is the major odour active compound present in the mine, being detected on 679 occasions throughout the mine, while hydrogen sulphide (H_2_S) and sulphur dioxide (SO_2_) were detected 5 and 26 times. Analysis of gas samples has shown that main DMS sources in the mine are coal extraction locations at longwall faces and development headings and that DMS is releasing during transport from main coal transport system. The dispersion simulations of odour sources in the mine have shown that the concentrations of DMS at median levels can represent relatively modest odour nuisance. While at peak levels, the concentration of DMS remained sufficiently high to create an odour problem both in the mine and on the surface. Overall, dispersion simulations have shown that ventilation regulation on its own is not sufficient as an odour abatement measure.

## Introduction

Coal (lignite) excavation at Coal Mine Velenje^1^ (CMV) occasionally emits unpleasant odours, which can affect a miner’s attention and hence safety awareness. Furthermore, fugitive odour emissions have a negative effect on the quality of life for local communities, which has become an increasing source of complaints. For these reasons, CMV has been carrying out research into techniques of controlling its odour emissions. This requires an understanding of the specific mining processes, identifying and quantifying the sources of odour and the odour active compounds responsible (Brattoli et al., 2014). Experience (the distinctive unpleasant smell to mine operatives) and historical gas concentration measurements have shown that the main odours at the mine are due to volatile sulphur compounds^2^ (VSC). The compounds with unpleasant usually have extremely low odour detection thresholds (μg/m^3^ range), which are many times lower than their toxic threshold limit value^3^ (TLV) in milligrams per cubic metre (mg/m^3^) range (Rosenkranz & Cunningham, 2003). The appearing of distinctive unpleasant smell which is detected by mine operatives and provokes complains of local communities is always connected with increased DMS concertation (monthly and additional control measurements) in the mine. Also, the distinct smell is similar as the one from released gas mixture of DMS and nitrogen from the gas cylinder.

CMV is one of the largest modern deep mines in Europe. It mines the largest Slovenian lignite deposit, which is one of the thickest known coal seams in the world. The seam is bowl shaped, 8.5 km in length, and 1.5–2.5 km wide with an average depth of 300 m (200–600-m deep) and extends almost under the entire Šaleška Valley (Si et al., 2015). The seam is on average 60-m thick with a maximum thickness of 165.8 m (Brezigar et al., 1987; Markič, 2009; Markič & Sachsenhofer, 2010). Over the past 147 years of operation, the mine has produced more than 252 million tonnes of coal with future plans to extract another 103 million tonnes.

The main VSC at CMV is dimethyl sulphide^4^ (DMS), while hydrogen sulphide (H_2_S) and sulphur dioxide (SO_2_) are less significant. The odour detection thresholds^5^ (ODT) of detected VSC’s are SO_2_ 2.32 mg/m^3^, H_2_S 0.58 μg/m^3^, and DMS 7.75 μg/m^3^ (Nagata, 2003).

DMS has a distinctive offensive smell similar to combination of decaying cabbage, seaweed, garlic, sulphur, and glue (Barczak, Możaryn, et al., 2022b; Fisher et al., 2018) and has negative hedonic characteristics (Qiao et al., 2011). At CMV, the presence of DMS was first noted in the late 1980s as an unpleasant odour when the access roadways for the upper NW part of the coal seam were being developed. Later, in the 1990s, DMS was once again encountered when CO gas sensors sounded without any visible indication of a significant oxidation process, which was later found to be due to the cross-sensitivity of the CO sensors to DMS. Currently, coal production is increasingly centred in this area, and odour complaints are expected to increase in the future. The origin of the DMS in the lignite seam is yet to be fully understood, but it is believed that it originates from the early stages of coal formation during the decay of organic matter (Kozinc, 2005). DMS is produced during the anaerobic microbial decomposition of methoxylated aromatic compounds present in the freshwater sediments (Finster et al., 1990) such as the lignite-bearing Pliocene sediments of the early Velenje Basin (Markič, 2009; Markič & Sachsenhofer, 2010). Therefore, it is believed that the DMS is retained during the rapid accumulation and burial of plant material and subsequent coal formation only to be released during coal extraction.

There exists an extensive body of literature concerning odour theory, including odour perception (Powers et al., 2004a; Powers & Corzangeno, 2004a), odour parameters (Nicolai & Pohl, 2005; Powers et al., 2004b; Powers & Corzangeno, 2004b; St. Croix Sensory. Inc., 2003), analytical methods (Conti et al., 2020; DEFRA, 2010; Gebicki et al., 2016; IPPC, 2002), olfactometric techniques (Conti et al., 2020; Gebicki et al., 2016; IPPC, 2002; McGinley & McGinley, 2003; Nicolai & Pohl, 2005; St. Croix Sensory. Inc., 2003), electronic nose (Gebicki et al., 2016; Karakaya et al., 2020; Kim et al., 2022), sampling and emission rate determination of odour sources (Boeker et al., 2010; Bylinski et al., 2019; Gebicki et al., 2016; Hudson, 2009; Juarez-Galan et al., 2010; Parcsi, 2005; Le et al., 2013; Trabue et al., 2008), monitoring methods (Benzo et al., 2010; Kost & Richter, 2010), and atmospheric dispersion modelling (Boeker et al., 2000; Capellia et al., 2013; Conti et al., 2020; Freeman et al., 2000; Li, 2009; Scire et al., 2000; Xing, 2006). Most studies, excluding those on odour theory, relate to industries that have well-defined odour nuisance issues, e.g. the agricultural and livestock industry (Koziel et al., 2010; Parcsi, 2005; Xing, 2006), waste water treatment plants (Barczak, Fisher, et al., 2022a; Freeman et al., 2000), municipal waste sites, recycling facilities, transfer stations, composting facilities (Fischer et al., 1999; Gebicki et al., 2016; McKendry et al., 2002), and industrial plants such as paper pulp mills, petroleum refineries, food processing, leather manufacturing, smelting of non-ferrous ores, steel mills, the manufacture of certain abrasives, paint manufacture, rendering, sulphur dioxide scrubbing, starch manufacturing (Kenneth et al., 2004) and tobacco factory (Zagustina et al., 2010), and biogas production (Vanek et al., 2015).

On the other hand, little information is available concerning fugitive emissions of odorous gases from mining activities in general. In addition, addressing potential odour issues as part of making an impact assessment is relatively new in planning coal mining projects. Odour dispersion modelling has been performed as part of an air quality impact assessment for a new ventilation shaft at the Illawarra coal mine, NSW, Australia (Kellaghan, 2010). For modelling purposes, the mine used cumulative odour measurements taken in the mine’s ventilation air from existing ventilation shafts. Gas compositional analysis revealed that the volatile organic compounds^6^ (VOC) were mostly below the limits of detection and did not pose an odour issue. Similarly, the Tasman Underground Mine (NSW, Australia), when seeking consent to extend their underground mine operations, also performed an air quality assessment on account that the development of the mine could potentially produce odorous emissions from the existing and proposed ventilation shafts (Kellaghan, 2012). No odour impact, based either on the levels of odorants in the ventilation shafts or in the actual coal seam, was detected. The Wilpinjong Coal Mine (NSW, Australia) analysed ambient air quality as a response to complaints by local residents (Cox & Isley, 2014). All of the active odour compounds were below the human odour detection threshold, and it is not clear from the study which odorants were responsible for the complaints. The Kanmantoo Copper Mine (SA, Australia) has performed a study for environment protection and rehabilitation while (PEPR, 2016) seeking consent for extending the life of open-pit mine for excavation and production of copper-gold concentrate. Odour monitoring results have confirmed the predicted odour dispersion model and showed that odour is not anticipated to result in negative impacts from the mining operations. The study of environmental impact of gold mines in Oman and the pollution impact by heavy metals (Abdul-Wahab & Marikar, 2012) also included odour measurements from water from nearby well used for irrigation. The odour from the water was not detected. Surprisingly, none of these studies included DMS in their analyses despite its low OTD.

For the assessment of potential health hazard due to the H_2_S emissions from the mine with underground copper ore deposit extraction (Kupczewska-Dobecka et al., 2015) also air samples and dispersion modelling were included. Room pillar technology is used to extract copper ore in the underground part of the mine. Mine ventilation is provided with four main fans with a nominal capacity of 400 m^3^/s each. For determination of H_2_S, 20 samples were collected in the diffusors, and 24 samples were collected in nearby settlements with distances between 2 and 5 km. Maximum measured concentration of H_2_S in the emitter was 286 μg/m^3^. In Poland, the reference values for H_2_S that are unlikely to cause adverse health effects in the general population are 5 μg/m^3^ (averaged over the period of the year) and 20 μg/m^3^ (averaged over 1 hour). At selected sites, H_2_S concentrations did not exceed 20 μg/m^3^ in 1-h air samples and 5 μg/m^3^ in 5-h air samples. Maximal modelled 1-h H_2_S concentration of 10 × 10 km^2^ area was 1.91 μg/m^3^. While there were no exceedances of health hazard H_2_S concentrations, there were regular complains of unpleasant sensation of smell from nearby settlements. According to the data obtained from the Ministry of Environment in 2013, as much as 1323 air pollution complaints were recorded in Poland and 65.7% referred to odour.

The literature research since 2011 point out that there are almost no published studies exist of DMS emissions from mining activities. Only published work referring DMS to mining activities is from oil sand mine operations in Fort McKay area in Alberta (Canada) and from those performed by CMV. In the air quality investigation of 30-km radius around Fort McKay of fife year monitoring period was performed due to the countless environmental complaints regarding oil sand mining activity in the area (AER, 2016). During monitoring period between 2010 and 2014 were 172 complaints and 165 were related to odours. Between 47 priority odorant candidates was also DMS. The investigation focused on six oil sand mines and one in situ facility. Selected were 16 continuous monitoring point and various others sampling points. Ambient air from sampling canisters of 1-h and 24-h samples was analysed for 60 volatile organic compounds and 20 reduced sulphur compounds. The DMS presence was analysed in 126 samples and was never detected (detection limit was 2.58 μg/m^3^).

The only published studies referring to DMS emissions from coal mining activities are those performed on coal from Velenje Coal Mine of gaseous sulphur emissions (COS, CS_2_, and DMS) from coal stockpiles (Kozinc, 2005; Kozinc et al., 2004) and a study on the levels of DMS in the return airway of a longwall face (Zapušek & Marsel, 1998), which were briefly summarised by Zhang (2013) in an IEA Clean Coal Centre report. The estimated daily emissions of COS and CS_2_ for the whole stockpile in the sampling period were 20 g of CS_2_ and 70 g of COS (gas concentrations were in μg/m^3^ range). The DMS concentrations fell to less than 2.58 mg/m^3^ within a few days as it is only released from freshly loaded coal. The measured DMS concentration levels in the mine air, which was sampled once a week for 15 consecutive weeks in the return airway of a longwall face during coal production, ranged from 55.47 to 128.48 mg/m^3^. During a series of in situ coal desorption tests made in 1998 and 1999 (5 boreholes and 10 desorption tests (Erico Ltd, 1998 and 1999)), DMS was detected in all but one sample (max. concentration 516 mg/m^3^), while H_2_S was < 1.42 mg/m^3^.

In the industrial or agricultural processes such as pulp and paper manufacturing, oil or petroleum refining, food decay, composting, landfilling, fish processing, sewage and wastewater treatment, leather manufacturing, paint, rendering plants, sulphur dioxide scrubbing, and starch manufacturing plants, DMS is a typical gaseous odour pollutant (Barczak, Fisher, et al., 2022a; Kenneth et al., 2004).

The removal or degradation of DMS odorant before exhausting into the atmosphere is of great significance to improve the local air quality. DMS has the unique capability of enhancing and intensifying other odours. Due to this property, it is used in warning odorants and odour masking agents (Kenneth et al., 2004).

DMS is also a substantial contributor of the aroma to some food items, such as beer (Stafisso et al., 2011), red wines (Lytra et al., 2016), truffles (Feng et al., 2019), many vegetables and fruits (tomatoes, sweetcorn, grapes, asparagus, and brassicas), honey (McGorrin, 2011; Schäfer et al., 2009), chewing gum (Kenneth et al., 2004), and cheddar cheese (Qian & Burbank, 2007).

One of most significant discharges of DMS and other reduces sulphur compounds (H_2_S, CH_3_SH, (CH_3_)_2_S_2_) can be from Kraft pulp mills (Kenneth et al., 2004). There are many sources in the mill. Some sources emit a small gas volume with high concentrations (blow heat recovery, turpentine recovery vent, evaporator hotwell vent, and foul condensate storage tank), while others have large volumes with low concentrations (brown stock washer filtrate, tanks, and hood; weak and strong black liquor storage tanks; knotter hood; black liquor oxidation vent; and contaminated condensate tanks). Typical DMS concentration of high volume source is 0.52 mg/m^3^ and for low volume source is 38,700 mg/m^3^.

Ambient air samples were collected at several locations in the community around a major Canadian pulp and paper plant over a period of several months, before and after major process changes (Catalan et al., 2007). In spring of 2006, they permanently closed one of two Kraft pulp mills on site and the shutting down of a chemical recovery boiler and associated black liquor oxidation systems. DMS was found to be the most abundant reduced sulphur compound in ambient air before the changes with an average concentration of 3.84 μg/m^3^. After the changes, the average concentrations of DMS decreased by 70%.

At landfill sites, over 300 trace compounds have been identified in landfill gas. Unpleasant odours are usually associated with the sulphur containing compounds, primarily mercaptans and sulphides. The vast range of trace compounds measured in landfill gas reflects both the anaerobic decomposition processes taking place in the waste mass and the wide range of chemicals introduced via the industrial and commercial waste streams (McKendry et al., 2002). DMS is a common odorant in landfill gas typically found in concertation range between 0.02 and 135 mg/m^3^.

The study of sulphur source from livestock production in Denmark exposes H_2_S as major sulphur source. Finisher pig production is estimated to be the largest source of atmospheric sulphur in Denmark (Feilberg et al., 2017). The only other sulphur compounds measured consistently in the ppb range are methanethiol and DMS, but these only constitute about 2–5% of H_2_S. Measurement campaigns were carried out over 6-year period from 2009 to 2015 on fife pig production facilities. The measured concentrations DMS were between 4.39 and 10.58 μg/m^3^.

In the study of identification of volatile sulphur odorants from 253 samples collected from emitted from ageing wastewater biosolids, whose production has been steadily increasing with commissioning of new wastewater treatment plants because of increasing population as well as more stringent effluent treatment and discharge standards, DMS was one of three VSCs (out of ten) that were identified by both analysis used in the study (GCSCD and GC-MS). The maximum DMS value was 4.53 × 10^3^ μg/m^3^ and was second highest maximum value after H2S whit value equal to 59.9 × 10^3^ μg/m^3^ (Barczak, Fisher, et al., 2022a).

The study in 2018, of identification of odour sources in two biogas plants in Poland, showed DMS concentrations up to 1.26 mg/m^3^ (Wiśniewska et al., 2019).

Physical-chemical and biological techniques are now available for removing odours from air streams including biofilters, biotrickling filters, membrane bioreactors, wet scrubbing, adsorption, and chemisorption, and more recently, methods based on photo-dissociation, electron beam irradiation, corona discharge decomposition, and catalytic and ozone oxidation (Qiao et al., 2011). However, DMS is one of the least biodegradable compounds among the odorous sulphur containing gaseous pollutants; consequently, it always needs improved systems out of the conventional biological setups. Traditional physical-chemical approaches to DMS removal mainly include wet scrubbing, adsorption, and chemisorption (Kenneth et al., 2004).

One of the largest available odour control systems designed to serve, for example, a water treatment plant, consists of a series of either biofilters or chemical scrubber units with capacities of up to 69.4 m^3^/s (ASK Piearcey Ltd, 2014). In underground coal mining, cumulative air flowrates are extreme and, at CMV, are between 340 and 420 m^3^/s. Clearly, existing systems could not possibly handle the large volumes of exhaust gases emitted from a coal mine, and either new or upgraded solutions must be developed. In addition, odorous mine gas emissions depend on many factors, including the natural characteristics of the coal, presence of odour active compounds in the coal seam, production and ventilation design, and coal production intensity. For these reasons, it is a challenge to predict their actual concentration.

The objectives of this study were to recognise and estimate the main odour sources in the mine and to construct ventilation model of CMV to perform model-based odour analysis.

This was achieved by taking into consideration the characteristics of mine ventilation, mine gateway system (airways), and estimated odorous emissions of mine sources in order to establish a better understanding of the sources, dispersion, and ventilation based control options to reduce the presence of odour active compounds released during the coal extraction process.

## Materials and methods

### Analysis of odorous gas emissions

As the first step in this study, the long-term monthly monitoring data of gas concentrations in the mine atmosphere were analysed in order to determine the main odorants in the mine, their source, and to estimate emissions.

Monthly chemical analysis of mine gases is carried out to control gas concentrations in mine air and includes the following gases: CH_4_, CO_2_, DMS, H_2_S, SO2, O2, CO, H_2_, NO, NO_2_, and N_2_.

The N_2_ content of the mine gas is the difference between the sum of the monitored gases and from 100% (Erico Ltd, 2008–2013). The concentration of CH_4_, CO_2_, DMS, and H_2_S was determined using gas chromatography. The test method PM 3.01 was used for CH_4_, CO_2_ (GC—FID), while the test method PM 3.02 was used to determine H_2_S and DMS (GC—FPD) (Erico Ltd, 2008–2013). The concentrations of O_2_, CO, H_2_, NO, NO_2_, SO_2_ were determined using a gas metre with a built-in electrochemical sensor (Echo d.o.o., Slovenia). Oxygen and CO concentrations were determined using the PM 3.03 test method, while the remainder were determined using the PM 3.04 test method. All three methods were developed by Erico—since 2017 is named Eurofins Erico (Erico Ltd, 2008–2013) and are granted by an accreditation body (i.e. Slovenian Accreditation).

These mine air samples were collected in 2-L Tedlar® sampling gas bags and analysed in the laboratory within 24 h (Erico Ltd, 2008–2013). The monitoring sites were selected systematically in such a way that all coal production activities can be controlled. Air samples were collected in the return airflows of all main work sites in the mine and in all main returns of mine ventilation. Samples were not collected simultaneous at specific monthly monitoring campaign.

The monitored gases were divided on odorous and unodorous gases. The limits of detection of odorous gases were DMS 2.58 mg/m^3^, SO_2_ 2.67 mg/m^3^, H_2_S 1.42 mg/m^3^, NO 1.25 mg/m^3^ and NO_2_ 1.91 mg/m^3^. Of odorous gases, NO and NO_2_ were never detected. Therefore, only monitored sulphur gases were analysed in the following.

Based on monthly monitoring data, the characteristic gas concentrations of mine odour sources were statistically determined for identified mine sources. For model entry values, two levels of mine odour sources were quantified: median sources (50th percentile) and peak sources (97.5th percentile).

Additionally, the odour activity values (OAV) were calculated considering characteristic concentrations and ODT (Fisher et al., 2018):

1 $$\textrm{OAV}=\frac{\textrm{Concentration}}{\textrm{Odour}\ \textrm{detection}\ \textrm{threshold}\ \left(\textrm{ODT}\right)}\ \left[/\right]$$

The OAV represents the relative importance of an individual compound in a usually complex odour mixture. The OAV is defined as the ratio of the concentration of a single compound to the odour threshold for that compound. Conceptually, the larger the OAV, the more likely that compound will contribute to the overall odour of a complex odour mixture (Bylinski et al., 2017; Parker et al., 2012). The odour concentration, usually expressed in odour units (OU/m^3^), is numerically equal to the dilution factor necessary to reach the odour threshold that is the minimum concentration perceived by 50% of population. According to European standardisation, 1 OU/m^3^ is defined as the amount of odorant that, when evaporated into 1 m^3^ of gas air at standard conditions, causes a physiological response from a panel (detection threshold) equivalent to that of n-butanol (reference gas) evaporated into 1 m^3^ of neutral gas (Brattoli et al., 2011). Considering the definitions of odour concentrations and OAV in case of air mixture with only one odorant, the values of odour concentration and OAV are equal.

### Odour modelling in the mine and simulation of odorous gas emissions

The purpose of odour modelling in the mine was to simulate dispersion of odorous gas emissions from its potential sources and to the surface under variable mine ventilation conditions and the characteristic concentrations of odour compounds.

The DMS concentration and odour modelling in the mine are based on mine ventilation model of VCM designed in Ventsim Visual™ software (Ventsim™). The software allows 3D graphical representation, simulated paths and concentrations of smoke, dust, diesel particles or gas for planning of emergency situations, short and long-term planning of ventilation, and the simulation of gas and aerosol concentrations (Ventsim™, 2013). Since the odour units (OU/m3) are the number of dilutions of an odorous air to the odour threshold in case of olfactory measurements of odour sources and in case of only single odorant (measured concentration of odorant and ODT), the software can also be adopted for odour dispersion simulations.

The software treats every component in the mine air in the same manner. The concentrations of gaseous compounds are diluted according to the dilution ratios of the return airways and their dilution at airways junctions. For each studied component, also decay mechanism can be modelled.

**Fig. 1:**
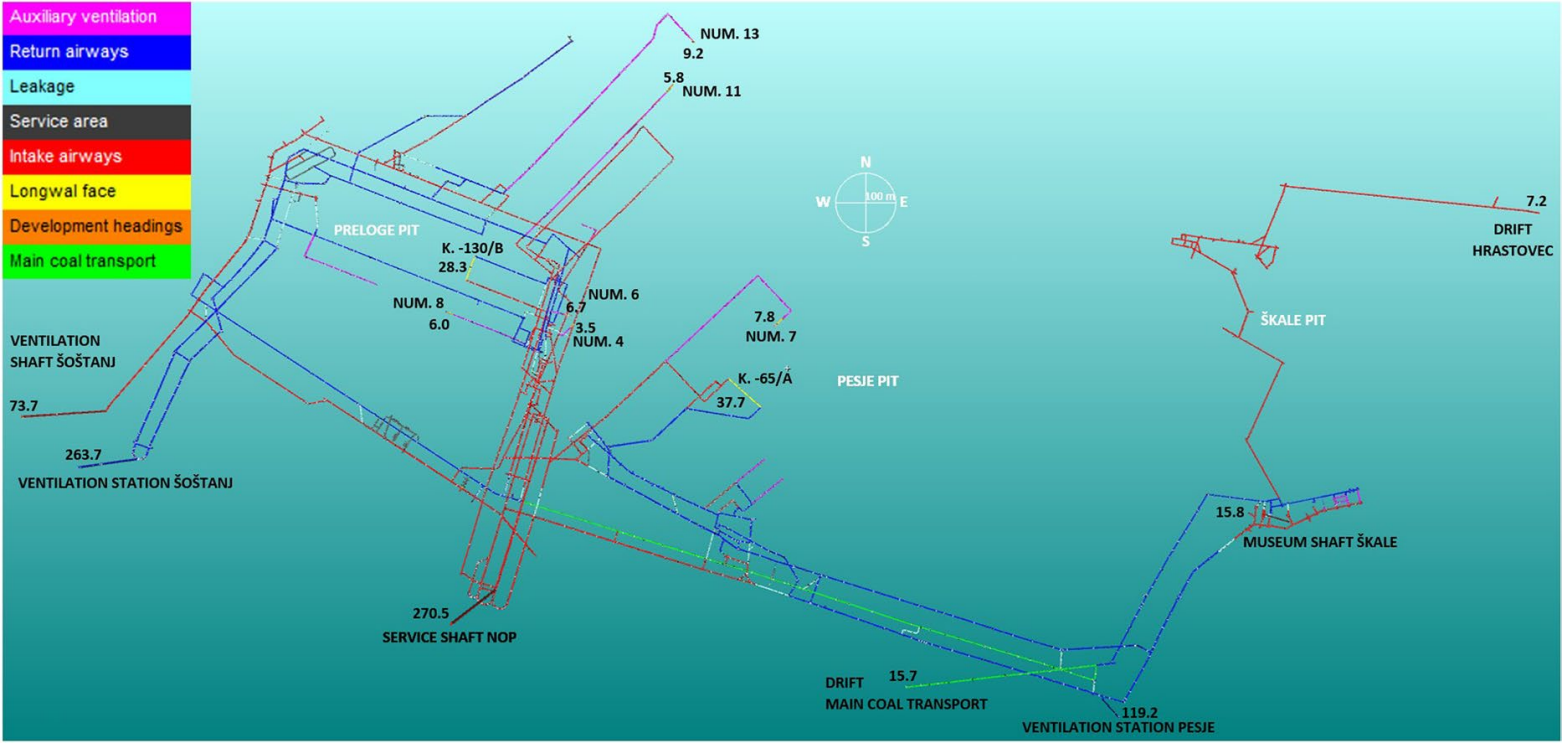
CMV plan modelled in Ventsim™ as of October 2012 with adjusted airflows (m^3^/s). The lines represent the actual gateways in the mine, and the colours of the gateways (legend in upper-left corner) show the different types of odour sources and the purpose of gateways regarding the mine ventilation

Designed ventilation/odour model is based on ventilation and mine data from October 2012 using monthly ventilation parameters for determination of air flowrate airways and represents the situation of mine ventilation in 57.85 km of underground facilities. Ventilation parameters are used each month to create a ventilation map of the VCM. The adjusted air flowrate of each airway was then determined by considering Kirchhoff’s first and second law theorems (McPherson, 1993). The mine ventilation map is a visual representation of the situation in the underground gateways (airways) and facilities with respect to adjusted airflows, direction of airflow, and locations of ventilation regulators: doors, barriers, boreholes, and shafts, auxiliary ventilators, and locations of gas sensors. The total resistances of the airways in the model are summarised from the “Zračenje” software developed in-house (Žibert, 2006). Figure 1, besides the mine gateway system, shows the locations of the main air intakes (service shaft NOP, ventilation shaft Šoštanj II, service shaft Škale, the main coal transport drift and Hrastovec drift), the main air exits—returns (ventilation stations Šoštanj and Pesje), the longwall faces (K-130/B and K-65/A), and the development headings: 4, 6, 7, 8, 11, and 13.

The deepest part of the mine is approximately 500-m deep. First, were used the whole data set to create a 3D model that defined every airway according to length, profile (round profiles were used for shafts, boreholes, ducts, and modified profiles for specific types of gateways used in CMV), cross-section, airway type, and total resistance. Other data included the average surface temperatures, both dry (10 °C) and wet (7 °C), together with temperature and atmospheric pressure (985 mBar). The network air density was calculated from the average monthly temperatures and pressures (1.20 kg/m^3^). In the model, fixed air flows were used for the upcast ventilation shafts (ventilation station stations), in the airways of the auxiliary ventilators and in the ventilation boreholes (all together 24 fixed flows). The model does not consider natural ventilation and compressible flows, which only have a significant effect when simulating mines deeper than 500 m (Ventsim™, 2013). All the other software settings were left as default.

In the second step, the air flowrate and directions of airways were modelled, iteratively adjusted for their total resistances and different ventilation regulation measures. To control the adjusted total resistances, were considered pressure drops on the main fans, which were 3460 Pa at the Šoštanj and 2040 Pa at the Pesje ventilation stations. The exhaust main ventilation at the VCM is provided by two main fans and a series of smaller auxiliary fans for ventilation of development sections or dead-end headings. The main fans are located at the Pesje and Šoštanj ventilation stations (Fig. 1). Each fan draws air up from the mine from five surface air intakes (situation as of 2012, Fig. 1). The main fan located at the Šoštanj station is a Turmag GVhv 31-1800 with nominal power 1800 kW (auxiliary fan: the same type), while at the Pesje station is installed a TLT-GAF 34-31 with nominal power 800 kW (auxiliary fan: Turmag GLH-28-660 with nominal power 600 kW) (Salobir, 2009a). The Šoštanj station provides approximately two-thirds of the required airflow rate, with the Pesje station providing the remaining one-third. The whole mine consists of 50–60 km gateways and facilities.

The general regulation of mine ventilation is possible by positioning the angles of main fan blades setup at each ventilation station. The airflow regulation potential with main fans of ventilation stations has direct effect on dilution potential of every gas or contaminant source in the mine. Main fan at Šoštanj has adjustable blades between angles −10 and +10°, while at Pesje, the main fan has blades that can be adjusted between -20 and +2°. The main fans’ characteristic curves (Salobir, 2009b) are customised in the Ventsim™ for the air flowrate simulation at Šoštanj: −10°, −8°, −6°, −4°, −2°, 0°, +2°, +4°, +6°, +8°, and +10° and for Pesje: −20°, −18°, −16°, −14°, −12°, −10°, −8°, −6°, −4°, −2°, 0°, and +2°. The setup of fan’s blades in October 2012 was +1° (Šoštanj) and −11° (Pesje). In the model, the airflow rates at Šoštanj ventilation station varied between 205.6 and 297.8 m^3^/s, and at the Pesje ventilation station, the airflow rates varied between 70.9 and 154.9 m^3^/s.

Verification of the model was based on the differences between the modelled and calculated air flowrate and between modelled and measured depressions of the main fans. The accuracy of the model, estimated on the basis of calculated and modelled airflows quantities in 226 airways, is ±0.07 m^3^/s. The modelled values of the main fan depressions were 3459.7 Pa at Šoštanj and 2040.4 Pa at Pesje stations.

Odour concentrations of mine odour sources for the model were calculated from characteristic DMS concentrations considering that 1 mg/m^3^ = 129 OU/m^3^ which calculated from DMS ODT 0.003 ppm (Nagata, 2003) and considered that 1 ppm = 2.583 mg/m^3^.

Odour emission rates (OU.m^3^/s) from the ventilation stations were calculated by multiplying the modelled odour concentrations (OU/m^3^) and airflow rate (m^3^/s).

## Results and discussion

### Analysis of odorous gas emissions and estimation of odorous sources

Over a 6-year period (2008/1–2013/12), 1028 point measurements were taken during mine operations. The monitoring sites were selected systematically so that the return airways of all production and development worksites and all main return airways were included. Under normal working conditions at the mine, H_2_S (released at the locations where the water is present in the mine, e.g. mine pump stations and SO_2_ (oxidation process in coal, e.g. mine fire) is rarely detected. Levels of SO_2_ throughout the mine exceeded the limit of detection on only 26 occasions (24 × 2.67 mg/m^3^, 1 × 5.34 mg/m^3^, and 1 × 10.68 mg/m^3^), while H_2_S was only detected 5 times (2.41–13.63 mg/m^3^).

The DMS was detected in 679 out of 1028 samples in levels of DMS between 2.58 and > 129 mg/m^3^ (levels above 129 mg/m^3^ were recorded as > 129 mg/m^3^). Out of all DMS detections, 116 times out from 163 measurements were detected on longwall faces, 262 times out of 398 measurements on development headings, and 30 times out of 72 measurements on main coal transport. The detection frequency shows that DMS is releasing during coal extraction process (also gateways are mostly built in coal seam) and DMS detection at main coal transport shows that DMS is released by desorption processes as the coal is being transported from the mine, similar to desorption from the coal in the stockpiles (Kozinc, 2005; Zhang, 2013) and VCM coal samples from boreholes in the coal seam (Erico Ltd, 1998 & 1999).

The previous researchers (Zapušek & Marsel, 1998) already identified DMS as major VSC in CMV. The analysis results confirmed DMS as the major odorant considering the detection frequency of VSCs and the releasing under normal working conditions. Considering the mine locations where the DMS were detected, the DMS mine sources were identified, and considering the DMS concentrations values, the DMS mine sources were quantified. The identified main mine DMS sources are the longwall faces (coal extraction working sites), the main coal transport system (system of rubber belt conveyors that is transporting coal directly to the surface), and the development headings (gateway building working sites). The analysis results of characteristic DMS concentrations and flowrates of DMS sources in return airways of main odour sources are presented in Table 1.

**Table 1:** Values of characteristic DMS concentrations with their OAV values and mass flows of DMS in the returns of mine odour sources

Odour/DMS Source		Longwall face		Main coal transport		Development headings	
Number of samples		163		72		398	
DMS concentration (mg/m^3^)	OAV	(mg/m^3^)	OAV	(mg/m^3^)	OAV	(mg/m^3^)	OAV
Max		129.0	16929	87.7	11512	129.0	16929
Mean		52.7	6910	7.9	1034	23.5	3081
Min		0.0	0	0.0	0	0.0	0
Std		55.1	7230	15.2	1990	34.5	4525
0.975		**129.0**	16929	**43.9**	5764	**129.0**	16929
0.75		129.0	16929	9.2	1210	30.3	3978
0.5		**31.0**	4063	**0.0**	0	**7.5**	982
0.25		0.0	0	0.0	0	0.0	0
0.025		0.0	0	0.0	0	0.0	0
Average airflow rate (m^3^/s)		**35.4**		**71.8**		**7.3**	
Median DMS source (mg/s)		**1097**		**0.0**		**55**	
Peak DMS source (mg/s)		**4569**		**3154**		**944**	

Bolded values 97.5th percentile and 50th percentile (median) are characteristic values of DMS concentrations considered for DMS sources estimation

Average airflow rates are characteristic values considered for DMS sources estimation

Last two rows of Table 1 are characteristic DMS sources considered in the model

The result shows great variations of DMS concentrations for each confirmed odour source, and also that DMS was present at sources between 44 and 71% of the time. For estimation of characteristic emissions of median DMS sources were taken into consideration median DMS concentrations at average air flowrates and for estimation of peak DMS sources were taken into consideration 97.5th percentile DMS concentrations at average air flowrates. Additionally, for the characteristic DMS concentrations, the OAVs were calculated, which shows dilution to threshold ratios. OAV values are very high (maximum OAV value was 16,929) due to the very low DMS’s ODT even while the DMS concentrations are at trace levels. Even lower ODT concentration as at DMS, resulted the OAV value of H_2_S at maximum measured concentration in 23,500. However, H_2_S was detected only in 0.5% measurements and DMS was detected in 66% measurements. The maximum OAV of SO_2_ was 5 and was detected in 0.6% measurements.

Longwall faces and development headings as odour sources are regarding ventilation and dispersion relatively simple if not considering the variability of production intensity and amount of DMS presence in the coal. While the main coal transport system of six successive conveyers of total length 2.6 km was ventilated with six intakes of fresh air at junctions and connection to the surface (Fig. 1) and, six leakage connections with main return airways are an odour source with very complex dispersion. All six leakages are dispersed to the ventilation station Pesje. Detected DMS in return airway of main coal transport in Preloge pit shows that it is being released during transport and that also all return airways—leakages in Pesje pit—must be considered.

**Fig. 2:**
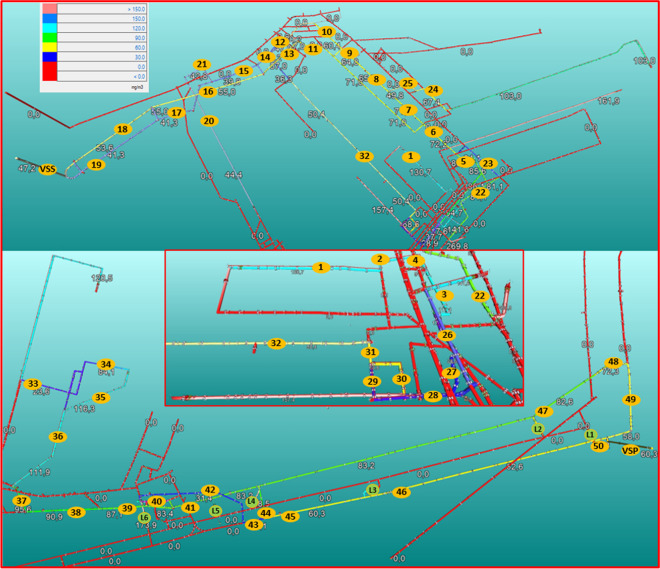
The monitoring location of simulations in Preloge pit (upper) and Pesje pit (bottom) with the simulated DMS concentrations at peak DMS sources. In the middle is presented enlargement of longwall panel K.-130/B area

For the main coal transport source in the model was considered that DMS is releasing at constant release rate from the constant mass flow of coal through the whole length. The source in the model was divided to each individual part of main coal transport airways accordingly to their lengths. Each individual part represented in the model as a partial source of DMS. The simulation results showed that 34.5% of main coal transport source was dispersed to the ventilation station Pesje and 65.5% was dispersed to the ventilation station Šoštanj which was detected with monthly measurements. Considering the whole main coal transport DMS emission rate at maximum concentration 87.7 mg/m^3^ and average airflow rate 71.8 m^3^/s than is 6297 mg/s and is potentially the biggest DMS source versus longwall faces (4569 mg/s) and development headings (944 mg/s). The division of whole source in the model at leakages from L1 to L6 was 3.5%, 1.8%, 6.1%, 6.7%, 6.7%, and 9.7%. Locations of leakages are marked on Fig. 2 (bottom figure) as a green circle.

### Simulations of DMS and odour concentrations

The odour model is based on DMS emissions. The model is considered inert since it assumes that all the sources of DMS do not decay over time.

It is known that DMS in the atmosphere does decay through reactions with photochemically produced hydroxyl (OH) and nitrate (NO_3_) radicals and ozone (O_3_) and nitrogen dioxide (NO_2_) (Kenneth et al., 2004 and Chen & Jang, 2012). A typical atmospheric half-life for DMS in the environment is from several hours to 3.5 days.

In addition, photochemical oxidation, although important on the surface, is not considered relevant in the underground mine.

At DMS study in the return airways of a longwall faces in CMV (Zapušek & Marsel, 1998), also the DMS stability was tested. A Tedlar gas sampling bag was filled with synthetic air (20% oxygen and 80% nitrogen) and with DMS standard with concentration of 51.3 mg/m^3^ and analysed every day by the gas chromatograph. During the analysis, gas standard in gas sampling bag was stored in dark place to avoid the UV-induced photodecomposition. The test results showed that DMS in Tedlar gas sampling bags is stable for at least 4 days.

During the study of effects of process changes on concentrations of individual malodorous sulphur compounds in ambient air near a Kraft pulp plant in Thunder Bay, Ontario, Canada (Catalan et al., 2007; Catalan et al., 2009), the stability of reduced sulphur compounds in the Teflon sampling bags was assessed.

A gas mixture also containing 3.87 μg/m^3^ of DMS was introduced in a clean Teflon bag and then periodically withdrawing aliquots which were analysed to monitor the changes in concentration over time. Any change from the initial concentration was due to decomposition of compounds in the gas phase or adsorption on the bag walls. The concentration of DMS was found to remain constant for more than 3.5 h. Similar results were obtained when the initial concentrations were doubled.

Additional details regarding upper two researches are presented in Table 2.

**Table 2:** Additional details from two researches regarding DMS stability over time

Paper	Purpose of citation	Sampling	Analytical analysis approach	Results
Zapušek and Marsel (1998)	DMS stability test	Laboratory test Sampling in one 2-L Tedlar bag of preprepared gas mixture from gas cylinder	GC-FPD Analysis every 24 h, starting at 0 h	After 120 h, the DMS concentration decreased to 48.0 mg/m^3^ and after 144 h decreased to 43.1 mg/m^3^ DMS stable for 4 days
Catalan et al. (2007) Catalan et al. (2009)	Stability test of reduced H_2_S, methyl mercaptan (CH_3_SH), DMS and dimethyl disulphide (DMDS)	Laboratory test One sampling Teflon bag with gas mixture containing 3.0 μg/m^3^ H_2_S, 9.4 μg/m^3^ methyl mercaptan, 3.9 μg/m^3^ DMS, and 3.9 μg/m^3^ DMDS Second sampling Teflon bag with doubled initial concentrations in gas mixture	GC-PFPD and interfaced with a cryogenic trap The absolute instrument sensitivity was 1.3 μg/m^3^ for H_2_S, 1.0 μg/m^3^ for methyl mercaptan, 0.24 μg/m^3^ for DMS, and 0.04 μg/m^3^ for DMDS During less than 7 and half hours, there were 7 analysis, starting at 0 h	All compounds remained within 10% of the initial concentrations for at least 3 h DMS was constant at least 3.5 h and DMDS for more than 7 h Similar results were obtained when the initial concentrations were doubled

Simulations of travelling (spread) times of DMS from the sources to the surface at operational air flowrate as of October 2021 showed that travelling times from longwall faces were between 556 and 750 s, from development headings were between 750 and 1836 s, and from main coal transport were between 521 and 1923 s. The longest travelling time 7409 s or 2.1 hours was from the main coal transport at simulation where the main fan blades at station Šoštanj was set on −10° (minimum air flowrate) and at station Pesje was set on +2°. The extreme travelling time is due to the changed airflow directions in some airways and dispersion through station Pesje instead of station Šoštanj as normal.

Based on the cases described above and modelled travelling times, any decay mechanisms of DMS were not considered in the model. The identified odour sources were the longwall faces K.-130/B and K.-65/A, road building faces 4, 6, 7, 8, 11, and 13 and the main coal transport system. In the model, sources are represented as point sources for each source, except at main coal transport, where the whole source in the model represents 7 point sources at return airway of main coal transport and at 6 leakages (Fig. 2, bottom figure).

For the study of DMS and odour dispersion analysis were considered 8 simulation scenarios, four for DMS and four for odour dispersion. Simulations tested dispersion of characteristic DMS and odour concentrations at median and peak levels and all characteristic concentrations at the operational air flowrate as of October 2012 and at maximum possible air flowrate due to the main fans’ characteristics (station Šoštanj at +10° and station Pesje +2°). The monitoring locations from 1 to 50 and ventilation stations Šoštanj (VSS) and Pesje (VSP) in the model (Fig. 2) were systematically selected to follow all dilutions in return airways from the sources and to the surface. The simulation results are presented in Table 3 and for simulation results of peak DMS concentrations at operational airflows in Fig. 2.

**Table 3:** The simulation results at the monitored locations

Monitored location	Operational airflows					Maximum airflows					Odour/DMS reduction
	(m^3^/s)	Median concentrations		Peak concentrations		(m^3^/s)	Median concentrations		Peak concentrations		
		(mg/m^3^)	(OU/m^3^)	(mg/m^3^)	(OU/m^3^)		(mg/m^3^)	(OU/m^3^)	(mg/m^3^)	(OU/m^3^)	(%)
1	35.0	31.3	4044	130.7	16,885	39.3	27.9	3604	116.3	15,024	10.9
2	37.3	29.4	3798	122.6	15,838	41.9	26.1	3372	109.0	14,081	11.2
3	39.0	28.1	3630	117.4	15,166	44.7	24.5	3165	102.2	13,203	12.9
4	50.4	22.0	2842	94.7	12,234	56.7	19.5	2519	83.8	10,826	11.4
5	61.8	18.4	2377	85.6	11,058	69.5	16.4	2119	75.7	9779	11.2
6	92.2	13.2	1705	72.2	9327	103.6	11.7	1511	64.1	8281	11.3
7	57.5	13.0	1679	71.0	9172	64.4	11.5	1486	62.9	8126	11.5
8	76.5	10.5	1356	65.7	8488	86.4	9.2	1189	57.9	7480	12.1
9	77.5	10.3	1331	64.8	8371	87.4	9.1	1176	57.2	7389	11.7
10	36.6	9.6	1240	60.4	7803	41.7	8.5	1098	53.2	6873	11.7
11	91.3	10.1	1305	59.1	7635	102.9	8.9	1150	52.1	6731	11.9
12	39.6	7.5	969	47.0	6072	44.8	6.7	866	42.1	5439	10.5
13	100.8	9.7	1253	57.0	7364	112.8	8.6	1111	50.5	6524	11.4
14	86.1	4.6	594	41.2	5322	96.3	4.2	543	37.4	4832	9.0
15	75.7	4.4	568	39.8	5142	85.4	4.0	517	36.1	4664	9.2
16	114.2	9.1	1176	55.0	7105	127.1	8.1	1046	48.9	6317	11.0
17	149.5	2.2	284	41.8	5400	166.0	2.1	271	37.6	4857	7.3
18	127.5	8.4	1085	53.6	6924	141.7	7.5	969	47.7	6162	10.9
19	136.1	2.2	284	41.8	5400	151.3	2.1	271	37.6	4857	7.3
20	71.1	0.0	0	44.4	5736	77.4	0.0	0	40.7	5258	8.3
21	73.8	0.0	0	42.8	5529	80.5	0.0	0	39.2	5064	8.4
22	11.6	4.7	607	81.1	10,477	13.1	4.2	543	72.2	9327	10.8
23	12.6	2.0	258	34.1	4405	13.7	1.8	233	31.5	4069	8.8
24	14.0	3.9	504	67.4	8707	16.2	3.4	439	58.3	7532	13.2
25	19.0	2.9	375	49.8	6433	22.0	2.5	323	43.0	5555	13.7
26	25.0	2.2	284	37.7	4870	27.6	2.0	258	34.1	4405	9.3
27	6.9	1.7	220	28.9	3733	7.8	1.5	194	26.3	3398	10.4
28	15.1	1.9	245	33.3	4302	17.4	1.7	220	30.0	3876	10.2
29	9.1	1.5	194	26.5	3423	10.7	1.3	168	22.7	2933	13.8
30	21.1	4.0	517	68.8	8888	23.4	3.6	465	62.7	8100	9.4
31	30.2	3.2	413	55.9	7222	34.1	2.9	375	50.2	6485	9.8
32	33.5	2.9	375	50.4	6511	37.8	2.6	336	45.2	5839	10.3
33	40.1	1.4	181	23.6	3049	53.3	1.0	129	17.7	2287	26.8
34	37.7	15.9	2054	84.1	10,865	50.4	11.9	1537	63.0	8139	25.1
35	46.9	24.5	3165	116.3	15,024	59.6	19.3	2493	91.7	11,846	21.2
36	49.2	23.4	3023	111.9	14,456	62.6	18.4	2377	88.1	11,381	21.3
37	57.6	20.0	2584	95.6	12,350	73.6	15.7	2028	74.9	9676	21.6
38	60.6	19.0	2455	90.9	11,743	80.4	14.4	1860	68.7	8875	24.3
39	63.3	18.2	2351	87.1	11,252	83.8	13.8	1783	65.8	8500	24.3
40	20.7	3.2	413	15.5	2002	26.6	2.5	323	11.7	1511	23.2
41	62.3	17.4	2248	83.4	10,774	81.8	13.3	1718	63.7	8229	23.6
42	25.2	2.7	349	31.4	4056	32.8	2.0	258	24.0	3100	24.7
43	27.2	2.5	323	29.1	3759	35.6	1.8	233	22.1	2855	26.0
44	30.1	15.3	1977	88.5	11,433	38.4	11.8	1524	68.1	8798	23.0
45	57.3	9.2	1189	60.3	7790	74.0	7.0	904	46.0	5943	23.8
46	59.9	8.8	1137	62.6	8087	77.4	6.7	869	47.8	6175	23.6
47	39.5	15.8	2041	82.6	10,671	51.9	12.3	1589	63.9	8255	22.4
48	45.2	13.9	1796	72.3	9340	57.6	11.1	1434	57.6	7441	20.2
49	56.3	11.1	1434	58.0	7493	72.0	8.9	1150	46.1	5955	20.2
50	62.9	8.4	1085	62.2	8035	81.4	6.4	827	47.5	6136	23.7
VSP	**119.2**	**9.7**	**1253**	**60.3**	**7790**	**153.3**	**7.5**	**969**	**46.9**	**6059**	**22.5**
VSS	**263.7**	**5.2**	**672**	**47.2**	**6098**	**293.0**	**4.7**	**607**	**42.5**	**5490**	**9.8**
Average		**10.0**	**1293**	**63.6**	**8212**		**8.4**	**1080**	**53.6**	**6922**	**15.4**

VSP (ventilation station Pesje) - the modeled values on the surface (all other monitored locations are in the mine)

VSS (ventilation station Šoštanj) - the modeled values on the surface (all other monitored locations are in the mine)

Average modeled values of monitored locations

The simulation results reveal a high odour concentration despite the low DMS concentrations also at median sources because of the low odour detection threshold of DMS. Odour concentration from median sources at ventilations stations means that the odorous emissions at ventilation station Šoštanj must be diluted to the odour detection threshold for additional 672 times by the atmosphere and at ventilation station Pesje for additional 1253 times. The odorous emission from ventilation station Šoštanj at odour concentration 672 OU/m^3^ and airflow rate 263.7 m^3^/s was 177,145 OU.m^3^/s, and odorous emission from ventilation station Pesje at odour concentration 1253 OU/m^3^ and airflow rate 119.2 m^3^/s was 149,370 OU.m^3^/s. While the emissions from Šoštanj are higher, the concentrations are lower due to the higher dilution rates of sources due to the 2.2 times higher airflow rate than in Pesje. For the longwall face K.-130/B in Preloge pit, the dilution rate to the surface was 9.3 and for the longwall face K.-65/A in Pesje pit was 3.2. Dilution ratios for development heading in Preloge pit were between 28.7 and 75.3, and in Pesje pit, only development heading num. 7 was dispersed to Pesje with a dilution ratio of 15.2. At median sources, main coal transport was not recognised as DMS/odour source as more the 50% DMS was not detected. The dilution ratio at peak sources were 3.7 in Šoštanj and between 29.8 and 91.7 in Pesje.

**Fig. 3:**
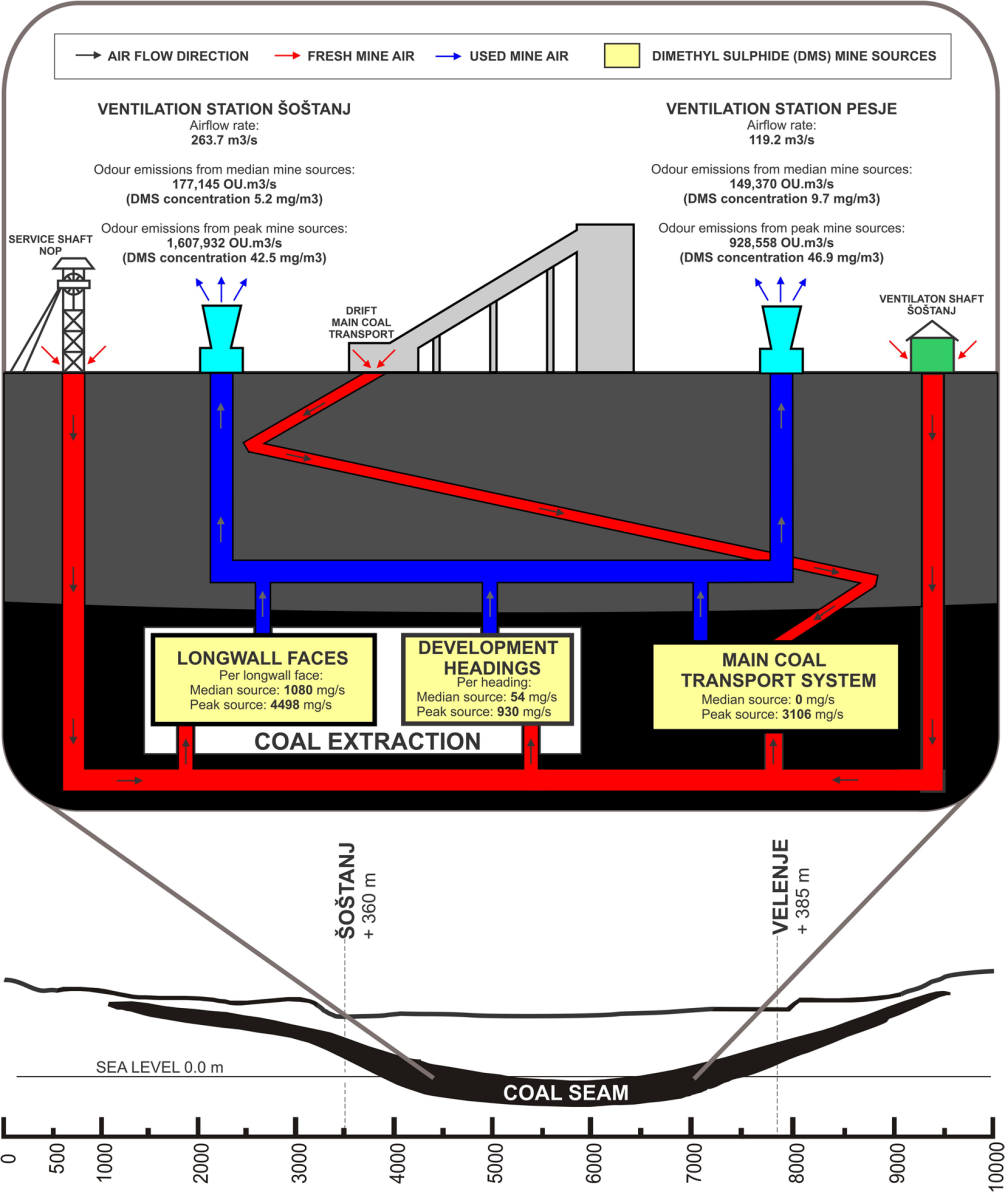
Graphical visualisation of main results of assessment of DMS odorous emissions during coal extraction process in CMV

In comparison, the odour emission modelling of newly planned ventilation shaft at the Illawarra coal mine, NSW, Australia (Kellaghan, 2010), showed that at an odour source equivalent to 219,500 OU.m^3^/s predicted that odour concentrations 3 OU/m3 would exceed only 1% of the time, what is in accordance with local odour regulations. In Slovenia, there are no odour regulations. If only level of odour emissions of CMV are compared, without taking into consideration the atmospheric conditions and vicinity of settlements, at the median odour sources, no odour complaints are expected. On the other hand, at peak sources what is considered as “worst case scenario”, the odorous emissions from Šoštanj with 1,607,932 OU.m^3^/s (at odour concentration of 5490 OU/m^3^ and an airflow rate of 293.7 m^3^/s) and from Pesje with (at odour concentration of 6059 OU/m^3^ and an airflow rate of 153.3 m^3^/s) 928,558 OU.m^3^/s are likely to lead to odour complaints. If considered scenario with only one longwall face at peak levels, the emission rate would be 580.242 UO.m^3^/s. Figure 3 presents a graphical visualisation of main results of characteristic DMS mine sources estimation and characteristic odour emissions on the surface.

Simulations of regulation of the main fans at peak levels to provide maximum air flowrate resulted in an overall additional reduction of concentrations for 15.4% on average. The ventilation reduction potential of concentrations regarding operational air flowrate as of October 2012 in Preloge pit was 10.6% and in Pesje pit was 22.9%. At peak sources, the average concentrations were 9.3 times higher than at median sources at monitored locations, and total emissions of peak sources were 7.8 times higher than of median sources.

The characteristic levels of DMS (Table 1) show great variations. At peak concentrations, longwall face source is 4.2 times higher than at median concentrations and at development heading source is 17.2 times higher. Main coal transport is not considered as DMS source at median levels and at peak levels is potentially the biggest source in the mine. The results indicate that DMS is not released only with extraction at longwall faces and development heading but is also releasing from the coal while is being transported to the surface. It is likely to be adsorbed on the lignite structure or trapped in the coal matrix, similarly as CO_2_ (Zavšek, 2004). From an adsorption/desorption study (Markič, 2009) of gases from different lithotypes of Velenje lignite, it was observed that the different lithotypes have significantly contrasting desorption properties related to differences in porosity. The specific surface area of pores in homogenous fine detrital lignite is more than 180 m^2^/g and 35 m^2^/g for xylite. Released DMS amount from sources greatly varies due to natural characteristics of the coal, presence of DMS in coal, production and ventilation design, and coal production intensity.

The odour concentration estimation of mine air is based on the DMS concentrations and its odour detection threshold. Simulation results show odour concentrations at ventilations stations between 672 and 7790 OU/m^3^. So far, rare separate point odour concentration measurements (according to standard EN 13725) at CMV’s ventilation stations were conducted. The monitored odour concentration levels were similar as modelled levels. Four separate odour concentration measurements at ventilation stations (NLHEF, 2017) in 2016 gave odour concentrations between 850 and 4500 OU/m^3^, and on 26 November 2007, three measurements (NIPH, 2008) showed odour concentrations between 3900 and 8400 OU/m^3^.

## Conclusions

There is almost no information concerning the fugitive emissions of DMS and odours in general from underground coal mining activities.

In this paper, this was addressed by describing and quantifying a dispersion of odours gases released from sources of CMV by focusing on analysis of gases monthly measurements in the mine and simulations of characteristic emissions of odorous compounds with mine ventilation model constructed in Ventsim™.

This research, based on the analysis of monthly gas measurements, confirmed DMS as a major potential odorant in the VCM released during coal extraction process and during the transport to the surface from identified and quantified mine odour sources: longwall faces, development headings, and main coal transport

The dispersion simulations of odour sources based on DMS concentrations in the mine show that median emissions represent relatively modest odour nuisance. While during peak emissions in the exit airways, odour is potentially high to be disturbing, and on the surface at the ventilation stations would be subject to odour complaints from the local residents. Simulating to additionally reduce odour levels with increasing air flowrate with the regulation of main fans showed that it is not an effective measure for mitigating odorous emissions, while measures by reducing coal production would impose severe economic penalties.

Since DMS is not regularly monitored in mines and levels are significantly varying due to its content and distribution in the coal, releasing mechanisms, mine ventilation design, and varying production intensity during the coal extraction process, the future work will focus on real-time monitoring of DMS levels and study of its correlations to coal extraction process to better understand and more accurately estimate odorous emissions from specific work phases of coal extractions process.

The DMS content in the seam is related to the petrographic heterogeneity of the coal; future research will involve investigating the coal desorption characteristics of DMS from coal.

In addition, to effectively address the odour issue at the VCM, especially in relation to fugitive odour emissions at the surface and for the design of technical measures for odour control, monitoring, and dispersion modelling of odour sources on the surface are necessary from hereafter.

However, underground coal mines are not widely recognised as an odour nuisance, and the development of technical abatement solutions to control odour from coalmining operations, especially given the large volume of ventilated air produced by the mine, will need more recognition of the problem and more support for its solving.

## Data Availability

The supplement data that support the findings of this study are available from Coal Mine Velenje (Premogovnik Velenje d.o.o.; https://www.rlv.si/), but restrictions apply to the availability of these data, which were used under licence for the current study, and so are not publicly available. Data are however available from the authors upon reasonable request and with permission of Coal Mine Velenje.

## Footnotes

1 CMV: Coal Mine Velenje (https://www.rlv.si)

2 VSC: volatile sulphur compounds

3 TLV: threshold limit value

4 DMS: dimethyl sulphide (CH_3_)_2_S

5 ODT: odour detection thresholds

6 VOC: volatile organic compounds
